# Clinical Characteristics and Prognosis of Influenza B Virus-Related Hospitalizations in Northern China during the 2017-18 Influenza Season: A Multicenter Case Series

**DOI:** 10.1155/2019/8756563

**Published:** 2019-11-07

**Authors:** Danyu Liu, Jun Xu, Xuezhong Yu, Fei Tong, Joseph Walline, Yangyang Fu, Kun Zhao

**Affiliations:** ^1^Department of Emergency, Peking Union Medical College Hospital, Chinese Academy of Medical Sciences and Peking Union Medical College, Beijing 100730, China; ^2^Department of Emergency, Second Hospital of Hebei Medical University, Shijiazhuang City, Hebei Province 050000, China; ^3^Accident and Emergency Medicine Academic Unit, Prince of Wales Hospital, Shatin, Hong Kong 999077, China

## Abstract

**Background:**

By weekly monitoring of China's influenza situation, Chinese National Influenza Center observed that the 2017-18 season was predominated by influenza B virus (IBV)/Yamagata. No studies regarding hospitalizations in adults with IBV infections have been performed. We aimed to describe the clinical characteristics of hospitalized patients with IBV infection in northern China.

**Methods:**

In this multicenter and retrospective study, we reviewed all consecutive adult patients with confirmed IBV infections at two level A tertiary teaching hospitals in northern China during the 2017-18 influenza season. Patients' clinical and diagnostic findings, as well as administered treatments and mortality data, were analyzed.

**Results:**

A total of 573 patients with a confirmed diagnosis of IBV infection were identified, of whom 22 cases were analyzed because of IBV-related hospitalization. Most patients were admitted to the intensive care unit (ICU) and had at least one underlying disease. The total in-hospital mortality was 27.3%. An elevated initial pneumonia severity index score, elevated direct bilirubin values, and lower platelet levels were associated with mortality (*p*=0.020, 0.013, and 0.049, respectively). The quick development of bilateral diffuse alveolar infiltrates was the most common imaging characteristics, following consolidation and pleural effusion(s). Risk factors such as HIV infection, pregnancy, underlying medical conditions, coinfections, and treatment delays were not associated with mortality.

**Conclusions:**

IBV should not be neglected because of its significant mortality. The elderly and patients with comorbidities, such as hypertension, diabetes, and connective tissue diseases, are more likely to have severe IBV-related pneumonia. Higher heart rates, direct bilirubin levels, initial PSI scores, and lower platelet levels are correlated with hospital mortality. Increased uptake in tetravalent influenza vaccine should be very helpful in preventing future cases of IBV hospitalizations.

## 1. Introduction

Influenza epidemics occur worldwide annually, but the 2017-18 season was one of the most severe influenza epidemics since the 2009 influenza A (H1N1) was pandemic in China [1]. There have been three influenza strains described in humans: A, B, and C, with influenza A being very common, B less common, and C very uncommon [2, 3]. The threat that influenza B virus (IBV) poses to human patients has been somewhat controversial in the past decade. In adults, IBV has been generally thought to be less pathogenic than influenza A virus (IAV). IBV usually can lead to mild self-limited respiratory infections [4]. However, other research studies concluded that IBV can cause similar rates of hospitalization and death as IAV in children [5, 6]. In addition, IBV infections in adults have been increasingly involved in sporadic severe cases [7–11]. As the Chinese National Influenza Center reported, the 2017-18 season was predominated by IBV (Yamagata lineage) and lower level circulation of IAV (H1N1 and H3N2) [12]. We aimed to describe the clinical characteristics and outcomes in hospitalized adult patients attributable to IBV in northern China during the 2017-18 season.

## 2. Materials and Methods

### 2.1. Study Design and Study Population

**Figure 1:**
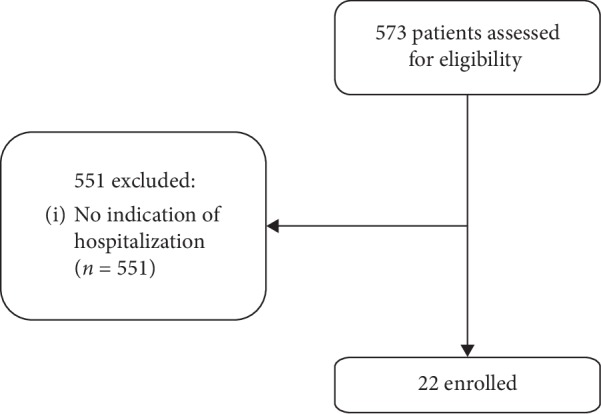
Recruitment flow chart.

This was a multicenter, retrospective review of all consecutive adult patients seen at Peking Union Medical College Hospital (PUMCH) and the Second Hospital of Hebei Medical University (SHHMU) during the 2017-18 influenza season. PUMCH and SHHMU are level A tertiary teaching hospitals in Beijing and Hebei Provinces, respectively. All patients with influenza-like symptoms were detected for IBV, and only those who were confirmed to be IBV-positive were screened. Influenza-like symptoms or diagnoses included a sudden onset of fever, dyspnea, acute exacerbation of chronic obstructive pulmonary disease, or pneumonia. Patients were evaluated by a qualified physician. Nasopharyngeal swabs were collected and tested for IBV by commercial real-time polymerase chain reaction (q-PCR) kits (Liferiver, Shanghai, China) following the manufacturer's instructions within the first 24 hours of admission. IBV infection was confirmed by the presence of influenza-like symptoms with a positive q-PCR test for IBV [13]. A total of 573 patients with a confirmed diagnosis of IBV infection were identified, of whom 22 cases were analyzed because of IBV-related hospitalization (Figure 1). Severe community-acquired pneumonia (CAP) was defined by the modified American Thoracic Society criteria or by reaching a risk class V of the Pneumonia Severity Index (PSI) [14]. This study was approved by the Ethics Committee Board of PUMCH and SHHMU (S-K539 and 2018-P026). The informed consent forms by individuals were waived because of the retrospective feature of this study.

### 2.2. Clinical Assessment

We used a standardized protocol to record clinical data. The following variables were recorded: demographics, CURB-65 index, initial PSI score and APACHE II score, underlying medical conditions, recent surgical history, smoking and alcohol habits, duration of hospitalization, clinical symptoms, vital signs, laboratory tests, chest radiography, hospital-acquired pulmonary infections, antiviral treatment, duration of symptoms onset to antiviral treatment, history of influenza vaccination, and overall outcomes.

### 2.3. Statistical Analysis

Continuous variables were expressed as a median with an interquartile range. Categorical variables were expressed as frequencies with percentages. The Fisher exact test for categorical variables and the Mann–Whitney *U* test for continuous variables were used to compare the differences between groups. Statistical analysis was performed using SPSS v. 24 (IBM Corp., Armonk, New York, USA), and a *p* value <0.05 was considered statistically significant.

## 3. Results

### 3.1. Demographics and Clinical Characteristics

22 patients with confirmed IBV-related hospitalizations were included during the study period. The patients' median age was 57 years (range, 49–70 years), and 40.9% were male (*n* = 9). There was evidence of underlying medical conditions in 63.6% of the patients (*n* = 14). These conditions included heart disease, lung disease, renal disease, liver cirrhosis, diabetes, hypertension, and connective tissue diseases. Five (22.7%) patients had smoking habits, 2 (9.1%) patients had alcohol drinking habits, and 3 (13.6%) patients had surgery less than a month before the onset of disease. No one was vaccinated for seasonal influenza during 2017-18 season. The median length of symptoms onset prior to hospitalization was seven days (range, 5–10 days). Symptoms at presentation included fever, cough, dyspnea, myalgia, rhinorrhea, chest pain, nausea, or seizures. Fever was reported in 20 (90.9%) patients, cough in 21 (95.5%) patients, dyspnea in 16 (72.7%) patients, chest pain in 6 (27.3%) patients, rhinorrhea in 3 (13.6%) patients, myalgia and nausea in 2 (9.1%) patients, and seizures in 1 (4.5%) patient. The median length of hospitalization was 13 days (range, 12–15 days). Demographic and clinical characteristics for survivors and nonsurvivors are detailed further in Table 1.

### 3.2. Diagnostic Findings

Diagnostic findings in survivors and nonsurvivors are described in Table 2. Most laboratory data were obtained within 48 hours of hospitalization, including arterial blood gas analyses, routine blood tests, liver and renal function tests, and creatine kinase (CK) levels. Only one patient showed baseline chronic abnormalities due to his underlying medical conditions. Eleven patients showed leukocytosis, but leukopenia was found in only two patients. Four patients had thrombocytopenia. The majority (81.8%) of patients had a decreased level of serum albumin (Alb), four patients showed an elevation of alanine aminotransferase (ALT), and five patients had an elevated level of creatinine (Cr). Laboratory abnormalities included lymphopenia (68.2%), thrombocytopenia (18.2%), and elevated direct bilirubin (DBil, 31.8%), lactate dehydrogenase (LDH, 81.8%), Cr (22.7%), and CK (31.8%). Of the 21 subjects who measured arterial blood gas, 17 (81%) had an oxygenation index under 300.

**Figure 2:**
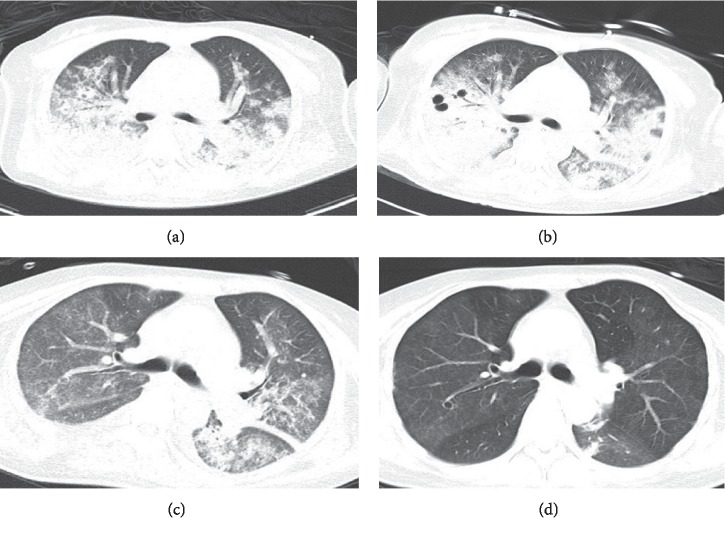
35-year-old woman with IBV-related pneumonia: (a) initial CT scan (day 10 after the onset of illness) shows diffuse alveolar infiltrates and consolidations in both lungs; (b) chest CT scan (day 17 after the onset of illness) reveals progression and widespread consolidations in both lungs; (c) consolidations in both lungs have begun to decrease by day 24 after onset; (d) consolidations in both lungs have further decreased by day 31 after onset.

**Figure 3:**
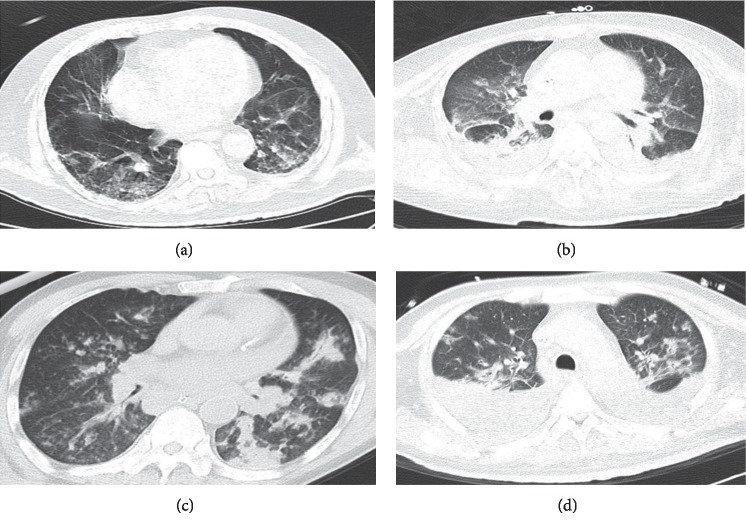
Chest CT findings in patients with IBV-related pneumonia. (a) 74-year-old man. Initial chest CT scan (day 10 after the onset of illness) shows bilateral diffuse interstitial infiltrates. (b) 64-year-old woman. Chest CT scan obtained on day 9 shows apparent consolidations accompanied by ground-glass opacities and pleural effusions. (c) 70-year-old man. Chest CT scan 9 days after the onset shows focal consolidations in the left lung. (d) 54-year-old man. Initial CT scan obtained on day 3 shows diffuse alveolar infiltrates with pleural effusion in both lungs.

All 22 patients underwent chest imaging on presentation and indicated the presence of pneumonia. Radiographic findings included bilateral infiltrates (18 patients) and unilateral infiltrates (four patients). The most common imaging findings were bilateral diffuse alveolar infiltrates in 15 cases and focal or lobar consolidation in 10 cases. Eight patients had pleural effusions on admission (Figures 2 and 3).

### 3.3. Treatment and Complications

Of the 22 patients identified, 20 (90.9%) patients received antiviral therapy. Of these 20 patients, 10 (50%) received antiviral treatment within 48 hours of hospital admission, but only one patient was treated with antiviral drugs within 48 hours of symptoms onset. Oseltamivir, peramivir, acyclovir, and ganciclovir are empirical antiviral drugs used in China [14]. Oseltamivir was given in all 20 subjects who were treated with antiviral drugs in this study. Acyclovir, ganciclovir, and peramivir were given together with oseltamivir in one, four, and five subjects, respectively. The median duration of symptoms onset before initiation of antiviral therapy was 10 days (range, 7–13). All 22 patients received empirical broad-spectrum antibiotics, with 90.9% of the subjects receiving combined antibiotic treatment. Adjuvant intravenous immunoglobulin was used in only one patient for 3–5 days, while corticosteroids were used in five patients for 3–5 days. Twelve (54.5%) patients required invasive mechanical ventilation, and 6 (27.3%) patients required vasopressors. Two patients were chosen for extracorporeal membrane oxygenation (ECMO) therapy.

Hospital-acquired pneumonia was identified in 15 clinical cases (68.2%), but there were no significant differences between survivors and nonsurvivors. *Acinetobacter baumannii* and *Aspergillus* were the dominant pathogens implicated. Fifteen of the 22 subjects had positive sputum cultures: ten of which were for *A. baumannii*, seven were for *Aspergillus*, three were for *Pseudomonas aeruginosa*, and one was for *Klebsiella pneumoniae*. Three patients had positive blood cultures: one was for *A. baumannii*, one was for *Burkholderia cepacia*, and one was for extended spectrum beta-lactamase-positive *K. pneumoniae*.

### 3.4. Clinical Outcomes

The majority (68.2%) of subjects were admitted to the ICU, and 13 (59.1%) patients fulfilled the criteria for severe CAP. The overall in-hospital mortality rate was 27.3% (six patients). Two patients died of respiratory failure caused by acute respiratory distress syndrome (ARDS). Four other patients died of multiple organ dysfunction syndrome (MODS), including septic shock and respiratory failure. The hospitalization time was shorter in nonsurvivors than survivors (median, 5 vs. 13 days, *p* = 0.010). There were no significant differences between the two groups in age, gender, underlying medical conditions, smoking history, drinking habits, or recent surgical history.

The median time of symptoms onset before hospitalization was not significantly different between survivors and nonsurvivors (median, 7 days, *p*=0.858). Clinical symptoms, such as fever, cough, dyspnea, myalgias, rhinorrhea, chest pain, nausea, and seizures, were similar in survivors and nonsurvivors. Laboratory findings showed a significantly higher heart rate (128 versus 101 beats/min, *p*=0.033), a lower platelet count (152 versus 231 10^9^/L, *p*=0.049), and a higher DBil (20.8 versus 3.2 mmol/L, *p*=0.013) in nonsurvivors.

Chest radiography was not significantly different in either group. Adjuvant corticosteroids had significantly harmful effects in survivors compared with the nonsurvivors in our study (6.3% versus 66.7%, *p*=0.009), but there were no significant differences between the two groups regarding antiviral therapy (87.5% versus 100%, *p*=1.0), early antiviral therapy (31.3% versus 83.3%, *p*=0.056), and adjuvant IVIG (6.3% versus 0%, *p*=1.0). The proportion of mechanical ventilation or vasopressors applied was similar between the two groups. Acute renal failure occurred in four (18.2%) patients. The median APACHE II score was 15 (range, 12–17). 63.6% of the patients had a CURB-65 score ≥2, and 72.7% had a PSI score at class IV or V. CURB-65 score ≥2, PSI class IV or V, and APACHE II score were not significantly different between the two groups, but the nonsurvivors had a significantly higher initial PSI score than survivors (109 versus 140, *p*=0.020).

## 4. Discussion

The clinical characteristics of IBV-related hospitalizations in adults have been poorly described in the past. This is the first study to do so based on data from the 2017-18 influenza pandemic season. Since the data we collected all came from a single season, we can avoid biases arising from season-to-season differences in strain virulence and population immunity.

Our study found that most patients fulfilled the criteria for severe CAP were admitted to ICU. However, whether illness severity is related to influenza types, subtypes, or lineages remains controversial. Caini et al. [15] found that except for the 2009 pandemic influenza A (H1N1) virus (pH1N1), the virus subtype did not seem to be a major determinant of severity in prior seasonal pandemics.

Demographics in our study were consistent with previous reports. The median age for our patients was 57 years, and most of the patients had at least one underlying disease. Although the median age of 57 is not particularly elderly, a higher percentage of the patients hospitalized with IBV who died were elderly. These findings support the idea that the elderly and patients with comorbidities are more likely to have severe influenza [16–18]. Many researchers have also suggested that asthma and chronic obstructive pulmonary disease (COPD) are the most common underlying conditions in patients hospitalized with seasonal influenza [19]. However, similar to the findings of Chiu [18], we found that hypertension, diabetes, and connective tissue diseases were the most common underlying diseases in patients hospitalized in northern China with IBV infections.

The clinical characteristics and disease development of patients in our study are very similar to pneumonia caused by other pathogens [14, 20, 21]. The presenting symptoms were not significantly different in survivors and nonsurvivors. The most common symptoms were fever and cough. Few patients had gastrointestinal symptoms, such as nausea or diarrhea. Acute influenza encephalopathy, most commonly reported in children, has also been described as one of the more rare complications of influenza virus infection [22]. However, the occurrence of seizures in just one (5%) of our patients is consistent with previous reports [23]. We found that patients who died had higher heart rates, DBil levels, and initial PSI scores but lower platelet levels than survivors. Leukocytosis is thought to be an unusual finding in influenza virus infections, with leucopenia being present in up to 25% of cases in some studies [23, 24]. However, leukocytosis was observed in 50% of the patients in our study, and leukopenia was very rare.

In contrast to the presentation of influenza A (H1N1) infections with a diffuse interstitial bilateral pattern, Gutierrez-Pizarraya et al. reported an alveolar pattern as the most common feature in 14 adults with IBV-related pneumonia [13]. Kato et al. reported chest computed tomography findings of bilateral diffuse ground-glass opacities and lung pathological findings of diffuse alveolar damage (DAD) in a case of severe respiratory failure associated with IBV infection [25]. However, our study found bilateral diffuse alveolar infiltrates and consolidation instead of interstitial infiltrates as the most common radiographic features in IBV-related pneumonia in adults. Thus, the imaging characteristics of IBV-related pneumonia may mislead the initial diagnosis and treatment for the IBV-infected patient.

Although the majority (90.9%) of patients in this study received antiviral therapy, only 5% initiated treatment within the recommended 48 hours of symptom onset. The median time of symptoms onset prior to hospitalization was seven days, which makes starting early antiviral treatment within 48 hours of symptoms onset quite difficult. Loubet et al. [26] observed that antiviral treatment was related to a reduced risk of ICU admission among subjects with underlying respiratory diseases rather than decreased mortality. As the US Centers for Disease Control currently recommends, antiviral treatment for all hospitalized, severely ill, and high-risk patients with suspected or confirmed influenza is an important adjunct to annual influenza vaccination. Antiviral treatment started within 48 hours of symptoms onset has been shown to benefit many patients [27]. However, antiviral therapy initiated after 48 hours can still be beneficial for some patients [28, 29].

The efficacy of adjuvant corticosteroids for CAP due to influenza virus infection remains controversial. Some investigations have suggested that early corticosteroid therapy for severe CAP can improve mortality [30, 31]. However, multiple studies have found that corticosteroids for influenza A (H1N1) pneumonia can lead to poor outcomes [32–34]. Although adjuvant corticosteroids did lead to poor outcomes in our study, we cannot infer that corticosteroids are harmful in IBV-related CAP because of the retrospective feature of our study.

The high-severity 2017-18 influenza season highlighted the importance of public health measures to help prevent influenza. Annual influenza vaccination remains the most effective way to protect against seasonal influenza and its potentially severe consequences [28]. As the influenza vaccine is not widely utilized in China, we are unable to show the clinical impact of influenza vaccination between our two study groups. Further education and popularization of the influenza vaccination is highly recommended and should be prioritized in pediatrics, obstetrics, and geriatric public health outreach efforts.

Risk factors, such as obesity, pregnancy, underlying medical conditions, and the presence of coinfections and treatment delays, were all related to increased severity in influenza A (H1N1) infection. Paddock suggested in 2012 that coinfection and cardiac injury contributed to fatal outcomes in influenza B infection [8], while Cohen found in 2014 that HIV infection was associated with IBV-related hospitalization [35]. However, we found that none of the patients were pregnant or had HIV coinfection in this study, and obesity was not included in our analysis because few obese patients were identified. For the other risk factors mentioned above, we found no differences in other underlying medical conditions, the presence of coinfections, or treatment delays between nonsurvivors and survivors. These results are consistent with other previous investigations [13, 36–38].

Our study has several limitations. First, our results cannot be suitable for all adults with influenza. There is selection bias due to only inpatients being included in this study. Second, the small sample size is due to the low incidence of IBV-related hospitalizations. While some studies have tried to combine data from several seasons to increase their sample size, we feel 22 cases was a relatively high number for a single season and worth examining on its own. Third, we cannot know the exact number of each lineage of the cases and which lineage is more likely to result in hospitalization in this study. The subtypes of IAV and lineages of IBV are detected by Chinese National Influenza Center instead of each hospital itself in China. Currently, IAV (H1N1) can also be detected in hospitals, so we believe that the lineages of IBV can also be detected in hospitals in the near future. Finally, the unequal number of patients in the two groups may result in bias in the results.

## 5. Conclusions

IBV should not be neglected because of its significant mortality. The elderly and patients with comorbidities, such as hypertension, diabetes, and connective tissue diseases, are more likely to have severe IBV-related pneumonia. Higher heart rates, direct bilirubin levels, initial PSI scores, and lower platelet levels are correlated with hospital mortality. Increased uptake in tetravalent influenza vaccine should be very helpful in preventing future cases of IBV hospitalizations.

## Figures and Tables

**Table 1:** Demographic and clinical characteristics and outcomes of IBV-related hospitalizations (comparison between survivors and nonsurvivors).

	All cases (*n* = 22)	Survivors (*n* = 16)	Nonsurvivors (*n* = 6)	*p* value
Age, years	57 (49–70)	57 (50–69)	61 (50–70)	1.000
Male sex (%)	9 (40.9)	6 (37.5)	3 (50)	0.655
Underlying medical conditions	14 (63.6)	9 (56.3)	5 (83.3)	0.351
Smoking history	5 (22.7)	3 (18.8)	2 (33.3)	0.585
Drinking habits	2 (9.1)	2 (12.5)	0 (0)	1.000
Recent surgery history	3 (13.6)	1 (6.3)	2 (33.3)	0.169

Clinical features				
Duration before hospitalization (days)	7 (5–10)	7 (5–10)	7 (4–9)	0.858
CURB-65 score ≥2	14 (63.6)	9 (56.3)	5 (83.3)	0.351
Initial PSI score	124 (87–138)	109 (83–133)	140 (123–147)	0.020^*∗*^
PSI class IV or V	16 (72.7)	10 (62.5)	6 (100)	0.133
APACHE II score	15 (12–17)	15 (12–16)	16 (15–19)	0.134
Duration before identification of influenza B virus (days)	11 (7–13)	13 (7–17)	8 (7–10)	0.203
*T*_max_ (°C)	39 (38–39.5)	38.5 (38–39.5)	39 (38.9–39.8)	0.367
Cough	21 (95.5)	15 (93.8)	6 (100)	1.000
Dyspnea	16 (72.7)	11 (68.8)	5 (83.3)	0.634
Myalgia	2 (9.1)	1 (6.3)	1 (16.7)	0.481
Chest pain	6 (27.3)	5 (31.3)	1 (16.7)	0.634
Rhinorrhea	3 (13.6)	3 (18.8)	0 (0)	0.532
Nausea	2 (9.1)	1 (6.3)	1 (16.7)	0.481
Seizure	1 (4.5)	1 (6.3)	0 (0)	1.000

Initial vital signs				
Heart rate (beats/min)	103 (96–126)	101 (90–116)	128 (115–135)	0.033^*∗*^
Respiratory rate (breaths/min)	26 (20–30)	25 (20–28)	31 (29–37)	0.115
MAP (mmHg)	94 (82.2–102.9)	96.8 (85.4–103.9)	86.7 (81.2–100.7)	0.494
PaO_2_/FiO_2_ ratio (mmHg)	183.3 (119.8–282.9)	183.3 (121.1–285.2)	173.1 (103.6–234.8)	0.541
Hospital-acquired pulmonary infections	15 (68.2)	9 (56.3)	6 (100)	0.121

Management				
Antiviral	20 (90.9)	14 (87.5)	6 (100)	1.000
Early antiviral (<48 h)	10 (45.5)	5 (31.3)	5 (83.3)	0.056
Adjuvant IVIG	1 (4.5)	1 (6.3)	0 (0)	1.000
Corticosteroids	5 (22.7)	1 (6.3)	4 (66.7)	0.009^*∗*^
Mechanical ventilation	12 (54.5)	8 (50)	4 (66.7)	0.646
Need for vasopressor	6 (27.3)	4 (25)	2 (33.3)	1.000
ECMO	2 (9.1)	1 (6.3)	1 (16.7)	0.481

Outcomes				
Duration of hospitalization (days)	13.0 (12.0–15.0)	13.0 (11.5–15.0)	5.0 (3.0–8.5)	0.010^*∗*^

CURB-65, confusion urea respiratory rate and age 65 scale; PSI, pneumonia severity index; APACHE II, acute physiology and chronic health evaluation II scale; MAP, mean arterial pressure; IVIG, intravenous immunoglobulin; ECMO, extracorporeal membrane oxygenation. ^*∗*^Statistically significant.

**Table 2:** Laboratory findings and chest radiologic characteristics of IBV-related hospitalizations (comparison between survivors and nonsurvivors).

Characteristics	All cases (*n* = 22)	Survivors (*n* = 16)	Nonsurvivors (*n* = 6)	*p* value
Laboratory findings				
pH	7.40 (7.34–7.46)	7.40 (7.34–7.46)	7.42 (7.32–7.45)	0.802
PCO_2_ (mmHg)	35.3 (30.7–53.0)	36.5 (31.6–35.3)	29.6 (24.3–40.1)	0.294
WBC (10^9^/L)	9.60 (5.60–17.80)	7.98 (5.60–15.90)	17.40 (5.96–25.12)	0.494
Lymphocyte (10^9^/L)	0.60 (0.19–0.91)	0.60 (0.18–0.97)	0.62 (0.40–0.78)	0.914
Hematocrit (%)	33.1 (29.6–41.4)	35.0 (29.3–42)	33.1 (30.9–37.7)	0.914
Platelet (10^9^/L)	177 (148–255)	231 (165–269)	152 (97–168)	0.049^*∗*^
ALT (U/L)	28.8 (14.3–41.4)	30.1 (13.6–41.3)	21.0 (15.3–40.3)	0.747
ALB (g/L)	28.8 (27.2–31.8)	28.5 (27.3–32.0)	30.0 (26.8–31.8)	0.971
LDH (U/L)	389 (341–625)	380 (309–513)	609 (473–625)	0.776
CK (U/L)	96 (66–371)	88 (66–245)	200 (84–506)	0.541
DBil (mmol/L)	4.8 (2.2–18.7)	3.2 (2.0–5.4)	20.8 (8.9–31.1)	0.013^*∗*^
Cr (*μ*mol/L)	71.5 (60.3–111.5)	67.0 (55.8–83.8)	97.5 (68.3–122.3)	0.294
Na (mmol/L)	139.5 (134.3–142.8)	140.4 (134.4–142.4)	137 (134–144)	0.914

Chest radiography				
Pleural effusion	8 (36.4)	6 (37.5)	2 (33.3)	1.000
Diffuse ground-glass opacities	5 (22.7)	4 (25)	1 (16.7)	1.000
Focal or lobar consolidation	10 (45.5)	7 (43.8)	3 (50)	1.000
Bilateral diffuse interstitial infiltrates	3 (13.6)	1 (6.3)	2 (33.3)	0.169
Bilateral diffuse alveolar infiltrates	15 (68.2)	11 (68.8)	4 (66.7)	1.000

WBC, white blood cell; ALT, alanine aminotransferase; ALB, albumin; LDH, lactate dehydrogenase; CK, creatine kinase; DBil, direct bilirubin; Cr, creatinine. ^*∗*^Statistically significant.

## Data Availability

The data used to support the findings of this study are included within the article.
